# Serious Games for Stroke Telerehabilitation of Upper Limb - A Review for Future Research

**DOI:** 10.5195/ijt.2020.6326

**Published:** 2020-12-08

**Authors:** Paula Amorim, Beatriz Sousa Santos, Paulo Dias, Samuel Silva, Henrique Martins

**Affiliations:** 1 Faculty of Health Sciences, University of Beira Interior, Portugal; 2 Institute of Electronics and Informatics Engineering of Aveiro; 3 Department of Electronics Telecommunications and Informatics, University of Aveiro, Portugal

**Keywords:** Serious games, Stroke, Telerehabilitation, Upper limb, Virtual reality

## Abstract

Maintaining appropriate home rehabilitation programs after stroke, with proper adherence and remote monitoring is a challenging task. Virtual reality (VR) - based serious games could be a strategy used in telerehabilitation (TR) to engage patients in an enjoyable and therapeutic approach. The aim of this review was to analyze the background and quality of clinical research on this matter to guide future research. The review was based on research material obtained from PubMed and Cochrane up to April 2020 using the PRISMA approach. The use of VR serious games has shown evidence of efficacy on upper limb TR after stroke, but the evidence strength is still low due to a limited number of randomized controlled trials (RCT), a small number of participants involved, and heterogeneous samples. Although this is a promising strategy to complement conventional rehabilitation, further investigation is needed to strengthen the evidence of effectiveness and support the dissemination of the developed solutions.

Stroke is a leading cause of disability worldwide. As mortality decreases, the number of survivors with several handicaps that benefit from rehabilitation has significantly increased (Lawrence et al., 2001).

Six months after stroke, 50% of survivors have hemiparesis and 26% have disruption of activities of daily living (ADLs) (Go et al., 2013). Although most of these disabilities will be permanent, rehabilitation programs implemented even years after injury are useful (Lloréns et al., 2015), improving quality of life and reducing the long-term expense of care in stroke survivors (Aminov et al., 2018). Patients can perform activities of daily living (ADLs) using only one limb, but this leads to a learned non-use phenomenon of the affected limb (Choi & Paik, 2018), progressive deterioration of motor function, increase of spasticity, joint stiffness, and more pain. Activation of the affected upper limb cortical area is beneficial in such cases.

The COVID-19 pandemic left many stroke survivors without rehabilitation and increased the use of telemedicine (Negrini et al., 2020). Telehealth technologies seem to be a promising tool, useful to enable consultations and service delivery from the acute phase to home and outpatient monitoring, to treatment, rehabilitation and prevention (Blacquiere et al., 2017). Compared to teleconsultations, rehabilitation program prescription of home exercises is much more challenging. The adherence to home exercises is usually low, whether due to fatigue, poor tolerance to effort, lack of motivation or musculoskeletal changes such as spasticity or joint limitations (Blacquiere et al., 2017). There is also need of reliable noninvasive monitoring procedures that, combined with self-management, could provide the necessary clinical data for reliable telerehabilitation (TR) (Pericas et al., 2013).

Telerehabilitation research started about 20 years ago (Jafni et al., 2017) with a focus on information technologies to provide distant support, assessment, and information to people who have physical and/or cognitive impairments (Schwamm Lh Fau - Holloway et al., 2009). The technology used includes videoconferencing platforms, wearable devices, audio and video communication, and social media, as well as many research-driven prototypes housed on a variety of platforms (Adamovich et al., 2009). More complex solutions incorporate robots (Garrett et al., 2018) and machine-learning-based systems (Lee et al., 2020). Of the several forms of TR possible approaches tested for stroke, videogame-driven TR approach is a promising one.

Serious games use VR for education, health, public policy, and strategic communication objectives (Zyda, 2005). It might be also a useful tool for stroke rehabilitation as it potentiates neuroplasticity at an early stage and stimulates sensorimotor areas that slowly deteriorate by disuse in a chronic phase (Adamovich et al., 2009; Levin et al., 2015). This approach also benefits pain therapy, depression treatment, and socialization after stroke (Castelnuovo et al., 2016; Maier et al., 2019; Mallari et al., 2019). The sensorimotor cortex of the affected hemisphere can significantly increase simply by the feedback of the contralateral hand by stimulation of mirror neurons (Calabrò et al., 2017).

Another mechanism is Motor Imagery Therapy, where the imagination of executing a movement of a segment promotes stimulation of the respective cortical area (Vourganas et al., 2019). VR-based serious games can activate the *nucleus accumbens*, with the release of dopamine, which is associated with reward-based learning, feelings of pleasure and motivation to perform specific behavior (Barrett et al., 2016).

This appears to be a safe approach. In experiments where VR has been used to train motor abilities, there have been no reported occurrences of cybersickness in impaired populations (Levin et al., 2015). Tracking technologies used in VR enables qualitative and quantitative assessment of stroke rehabilitation exercises (Lee et al., 2019) which are, otherwise, difficult to measure in home-based exercises. Machine Learning research using VR technology could develop intelligent decision support systems to identify salient features of assessment using reinforcement learning to assess the quality of motion and summarize patient specific analysis (Lee et al., 2020). Compared to conventional rehabilitation, serious games also have the advantage of promoting motivation (Dias et al., 2019).

This work aimed to review and analyse the clinical research on upper limb stroke telerehabilitation using serious games. The final objective was to identify limitations and opportunities as well as provide avenues for future research.

## REVIEW MATERIALS AND METHODS

This review is based on research material obtained from PubMed and Cochrane until April 30, 2020. The search terms included («virtual reality» OR «serious games» OR «exergames») AND «stroke» AND «telerehabilitation». Only 75 records were identified through database searching (53 articles in PubMed and 22 in Cochrane). In addition, reference lists of relevant reviews were searched by hand and four more records were added.

**Figure 1:**
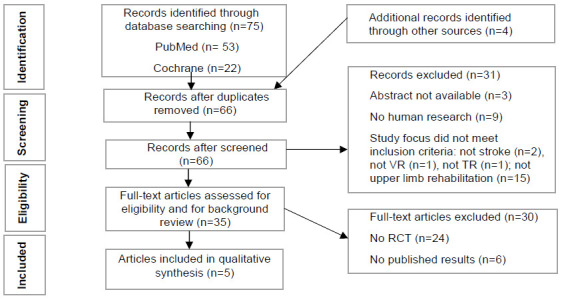
Flow Diagram Adapted from Preferred Reporting Items for Systematic Reviews and Meta-Analysis (PRISMA)

*Note.* This diagram shows the process for identifying and screening the articles for inclusion and exclusion.

The review was conducted and reported in accordance with the Preferred Reporting Items for Systematic Reviews and Meta-Analysis (PRISMA) statement (Moher et al., 2009) (Figure 1).

The question generated using the PICOS (Saaiq & Ashraf, 2017) components (Population, Intervention, Comparison, Outcomes, Study Design) was: «in stroke patients (P) are serious games for telerehabilitation (I) effective in upper limb rehabilitation (O) when compared with conventional rehabilitation (C)»? For qualitative synthesis, only randomised controlled trials (RCT) were included (S), as they are regarded as the best study design to test the efficacy of clinical intervention.

After removing duplicates, authors independently analysed titles and abstracts of 66 papers. Disagreements were resolved through review team discussion. After this procedure, the review included 35 studies that met inclusion criteria: (1) involving stroke survivors; (2) describing a rehabilitation intervention using telerehabilitation and virtual reality in form of serious games; (3) focusing on upper limb rehabilitation; (4) human research; and (5) written in English.

To analyse the clinical evidence only RCT with published results (n=5) were included. In order to analyse the quality of the included RCT, the first author used the Physiotherapy Evidence Database (PEDro scale) (de Morton, 2009) and discrepancies were resolved through discussion with co-authors. The PEDro scale was developed by the Physiotherapy Evidence Database to determine the quality of clinical trials. The PEDro scale consists of a checklist of 11 scored yes-or-no questions identified in Table 3.

## REVIEW RESULTS AND DISCUSSION

The review with the PRISMA method retrieved 35 results identified in Table 1.

**Table 1:** Description of Articles Initially Included by PRISMA Methodology

Article Type	Focus	Reference
Narrative review/scoping review	VR for rehabilitation VR for motor rehabilitation VR for upper limb rehabilitation in neurological disorders VR and TR for stroke rehabilitation VR and TR for upper limb rehabilitation	(Levin et al., 2015) (Holden, 2005) (Adamovich et al., 2009) (Putrino, 2014; Veras et al., 2015; Veras et al., 2017) (Brochard et al., 2010)
Systematic review	TR for stroke rehabilitation	(Chen et al., 2019)
Meta-analysis	VR for stroke rehabilitation	(Maier et al., 2019) (Karamians et al., 2020)
Discussion paper/opinion article	VR for rehabilitation VR and TR for motor rehabilitation	(Burdea, 2003; Garrett et al., 2018) (Piron et al., 2002)
Case study	VR and TR for stroke rehabilitation VR for upper limb rehabilitation after stroke	(Pareto et al., 2011) (Ding et al., 2018)
RCT protocols only (without published results)	VR and TR for stroke rehabilitation VR and TR for upper limb rehabilitation after stroke	(Sheehy et al., 2019) (Fluet, 2016; Gauthier et al., 2017; Kairy, 2015, 2018; Kairy et al., 2016) (Fluet, 2019)
RCT with published results	See Table 2	See Table 2
Other trials (not randomized and/or not controlled)	Augmented VR and TR for upper limb rehabilitation after stroke VR for rehabilitation VR for stroke rehabilitation VR and TR for upper limb rehabilitation after stroke	(Kuttuva et al., 2006; Piron et al., 2004) (Yang et al., 2018) (Lee et al., 2016; Valdés et al., 2018) (Chiu et al., 2018; Dias et al., 2019; Triandafilou et al., 2018)

Although reviews, systematic reviews, and meta-analyses of telerehabilitation after stroke have found either better or similar effects when compared with conventional in-person therapy, they have noted high heterogeneity in treatment content across studies (Chen et al., 2015a, 2015b; Lin et al., 2018; Sarfo et al., 2018). Some studies reported that TR is less expensive, but more studies are needed to draw more definitive conclusions (Barak Ventura et al., 2019).

VR shows moderate to strong evidence for efficacy (Adamovich et al., 2009), being enjoyable, and improving motivation and adherence (Valdés et al., 2018). VR has the advantage of monitoring patient movements and allowing activities that are unsafe to practice in the real world, such as crossing the street, driving, or preparing food. In a recent meta-analysis VR appears to be more effective than conventional rehabilitation, improving 28.5% of the maximal possible improvement of upper extremity functionality (Karamians et al., 2020). In constraint-induced movement therapy for rehabilitation of upper limb hemiparesis, which leads frequently to patient frustration in conventional rehabilitation, gamification through serious games seems to promote better acceptance by patients (Sheehy et al., 2019).

Besides VR serious games, augmented reality, which superimposes a computer-generated image on a user's view of the real world, also seems to have benefits in upper limb functionality training (Burdea, 2003).

In terms of efficacy, the purpose was to reveal non inferior solutions with a VR approach as compared with conventional therapy. A systematic review and meta-analysis showed that the outcome of stroke rehabilitation using game-based therapy could significantly outperform conventional therapy (Lohse et al., 2014).

From the review of articles included in Table 1 the authors identified features of serious games that are useful and practical for stroke rehabilitation. They should be easily portable to allow treatment anytime/anywhere, provide immediate feedback to the patient, and allow the monitoring of performance and progression in a quantitative and easily accessible way for the doctor/therapist (Proffitt & Lange, 2015). When evaluating serious games for rehabilitation, additional factors must be considered:

***Attention:*** Training protocols can incorporate multisensory feedback (auditory/visual) as well as performance feedback on unwanted motor compensations. Some systems adopt force feedback, which employs haptic or exoskeleton devices (Kato et al., 2015).

***Disability adaptation:*** Commercial gaming systems are designed for able-bodied participants and may not consider the physiological, motor, and cognitive aspects of recovery in rehabilitation. Designed with help of rehabilitation scientists, they may also lack the scalability of purpose-designed virtual environment systems (i.e., serious games) (Green & Wilson, 2012; Valdés et al., 2018). If the game difficulty is set too high, it can lead to frustration and abandonment by players with disabilities. Another limitation is the possible inadequacy of devices used to mediate patient's interaction with spastic hands. For instance, Nintendo Wii® game controllers or Microsoft Kinect® are challenging to use by any stroke survivor without adaptation (Burdea et al., 2003; Burdea et al., 2019).

***Adaptation to Rehabilitation:*** Serious games must meet the four basic principles of post stroke rehabilitation - intensive, task-orientated, provide feedback to the patient and motivating (Covarrubias et al., 2015).

***Data logging:*** with serious games it is possible data collection during periods of home exercise and remote patient monitoring (Lohse et al., 2014). Cybersecurity and data protection must be assured.

***Engagement:*** The principles of game design include meaningful play, feedback, goals, reward, challenge, difficulty, failure and flow; several studies suggest that gamification increases engagement in rehabilitation exercise (Barak Ventura et al., 2019; Burke et al., 2009; Rizzo & Kim, 2005). According to the goal-setting theory, the patient's engagement can be increased through setting small goals or targets that are realistic, manageable, and customized for the patient. However, the activities also need to be challenging enough for the patient to be engaged (Vourganas et al., 2019). Pleasant and interesting scenarios support the performance of ADLs, including skills in the kitchen, reaching objects with the paretic arm, crossing streets, shopping, and social interaction. Despite the image of a smile or the phrase “well done”, a sound and the knowledge of the score provide additional motivation (Dias et al., 2019).

***Level adaptation (Scaffolding):*** Another method of game mechanism is referred to as scaffolding. After a participant masters a new motor skill, another new challenge is introduced into the game. The player learns how to safely perform and practice the new skill. The player must master this skill before moving onto the next skill (Fluet, 2019; Fluet et al., 2019). Therapists must be able to calibrate the degree of difficulty. In this regard, artificial intelligence brings benefits to TR related to the speed of adaptation (Fluet, 2019).

***Socialization:*** Serious games promote socialization when played by multiple users on cooperative or competitive virtual tasks (Thielbar et al., 2020).

***Cost:*** Video games have been applied to upper limb rehabilitation since the 1980s. With the advent of less expensive and higher-fidelity technology, acceptance and clinical validity of games for serious purposes have been increasing (Barrett et al., 2016; James et al., 2018). Non-immersive or mobile VR do not require high-level graphic performance or expensive hardware, making them good candidates for providing low-cost, ubiquitous, and interesting treatment programs (Choi & Paik, 2018).

Adapting existing gaming frameworks as treatment protocols, rather than developing games from scratch, should be considered to foster richer gaming experience (Putrino, 2014).

When combined with remote monitoring (TR), the benefits of serious games are further strengthened (Garrett et al., 2018). This combined approach has been increasingly investigated (Green & Wilson, 2012).

Despite the potential, systematic reviews and meta-analysis addressing VR and TR for rehabilitation after stroke show that most of the studies had limitations. A reduced number of participants resulted in limited statistical validation (Aramaki et al., 2019; Ayed et al., 2019; Chen et al., 2015b; Laver et al., 2020; Laver et al., 2017; Maier et al., 2019; Mekbib et al., 2020; Viñas-Diz & Sobrido-Prieto, 2016). Very few studies have systematically addressed stroke patients' requirements in the home environment (Chen et al., 2019) and almost none were designed with direct input from patient and provider end-users (Birckhead et al., 2019).

Measuring the effectiveness is crucial to assess the benefits of TR and VR on stroke rehabilitation. The primary outcome should be reported before and after the intervention and between study arms with accompanying 95% CI (Birckhead et al., 2019). Yet, there is no consensus on what measures to employ to facilitate comparisons across interventions. Many different range of outcome measures have been used (Veras et al., 2015; Veras et al., 2017). The focus is often the functionality of the upper limb, balance, and postural control. Less common is the evaluation of daily functioning, independence, quality of life, pain, depression, fatigue, and socialization, despite their relevance for health quality. The choice of adequate measures is crucial to assess the quality of care, identify gaps, and define priorities in stroke care (Veras et al., 2017).

Satisfaction is another important indicator of the efficacy of therapeutic interventions. A high level of satisfaction promotes the patient's motivation to engage in rehabilitation (Chen et al., 2020; Piron et al., 2008).

Less used in this context is the holistic approach of the International Classification of Functioning (ICF) (Stucki et al., 2007), which highlights the importance of the environmental and personal factors in the health of individuals from the perspective of their influence on Body Function and Structure, Activity, and Participation. The effects of VR on stroke rehabilitation based on the ICF framework in Body Function and Body Structure are most frequently measured, mainly through the Fugl-Meyer Test, Action Research Arm Test, Ashworth Assessment scale, and manual muscle power testing. Activity and Participation (Aminov et al., 2018) are less often assessed, though these features are proposed as vital when designing game systems for stroke survivors. Published survey papers, taxonomies and framework are unanimous about the need for promoting socialization (Palma et al., 2017; Veras et al., 2017); (Palma et al., 2017).

The results of VR-TR intervention were positive in all five studies with benefits for the patients. However, an analysis of the RCT included in this review (Table 2), indicates that all were preliminary studies with a limited number of adult participants. The information about the age at which the stroke occurred were not always available, nor was information about the severity and type of stroke. There was no homogeneity in inclusion and exclusion criteria. One RCT (Piron et al., 2008) used VR therapy in control and experimental groups and TR only in experimental groups; the others compared a VR and TR approach with conventional therapy. In one study (Piron et al., 2008) the experimental VR-TR group did not include conventional therapy; in other studies, both experimental and control groups received conventional therapy, perhaps causing a confounding bias. The TR component was not always clear. The authors assumed that all five studies used TR to monitor the results of VR intervention; however, two studies (Choi & Paik, 2018; Piron et al., 2008) didn't specify how. The VR intervention was diverse. Rodriguez-de-Pablo et al., (2015) used a robotic system with a set of VR serious games. Fluet et al., (2012) and Fluet et al., (2019) used a Leap motion controller to capture patient movements while Choi and Paik (2018) used a smartphone with built-in sensors. Piron et al., (2009) used a virtual reality-based system delivered via the Internet. Each study used a different intervention protocol, including different therapy intensity and duration. To measure outcomes most studies measured upper limb functionality with the Fugl-Meyer Assessment. Only one study assessed patient motivation (Fluet et al., 2012) and just one assessed patient satisfaction (Piron et al., 2008).

**Table 2:** Description of RCT Included by PRISMA Methodology in This Review

Authors/Year	Participants	Intervention	Outcomes measurement	Results
Choi & Paik, (2018)	n=24 (n=12 in experimental group) Adults Ischemic stroke No information about time since stroke, nor stroke severity.	Experimental group received 30 min of conventional therapy and 30 min of the mobile game-based VR upper extremity rehabilitation program using a smartphone with built-in sensors and a tablet PC. Four simulations with several levels of difficulty. Level of difficulty of the game applications were individually adjusted. Control group received conventional therapy alone for 1 hour per day. Both groups received a rehabilitation program of 10 sessions of therapy, 5 days per week, for 2 weeks.	Fugl-Meyer Assessment of the upper extremity (FMA-EU) Manual muscle power testing Brunnstrom stage Satisfaction questionnaire (5 point Likert rating)	Results were presented in a descriptive way, without statistical analysis. Results suggested a greater improvement in the FMA-UE, B-stage, and manual muscle power testing post treatment with the mobile game-based VR upper extremity rehabilitation program than with conventional therapy. The effect was maintained until the one-month follow-up. Patients in the experimental group responded positively to the feasibility questions (display of the program: 4.08±0.62; readability of the program: 4.25±0.62 and the convenience of program usage: 4.08±0.67)
Fluet et al., (2019)	n=11 (n = 5 in enhanced group) Adults The severity of cognitive changes and the functionality of the upper limb and of neglect were part of inclusion criteria. No information about time since stroke nor type of stroke (ischemic/hemorrhagic).	Enhanced motivation group (EM) trained using a system integrating a Leap Motion controller, a passive arm support, and a suite of hand rehabilitation. Three simulations provided 8–12 levels of difficulty and complexity, and were individually adjusted. Graphics and scoring opportunities increased at each new level. Unenhanced control group (UC) performed the same simulations. Difficulty was increased incrementally utilizing an algorithm, making adjustments imperceptible. Both groups practiced ^«^as much as possible,” at least 20 minutes daily for 12 weeks.	Fugl-Meyer Assessment of the upper extremity (FMA-EU) Box and Blocks Test (BBT) Time and duration of training Intrinsic motivation inventory	Student's t-test for pretest to post-test score change was significant for the BBT, for the entire group (p = 0.0485) and for FMA-EU (p < 0.001) EM group: averaged 95 – 95 minutes of training per week, range was between 40 and 276 minutes. UC group: averaged 35 – 31 minutes of training per week, range was between 3 and 93 minutes. Intrinsic motivation levels were better for the EM group and motivation levels were maintained for the 12-week protocol.
Piron et al., (2009)	n=36 (n=18 in experimental group) Adults 7-32 months after stroke No information about stroke severity nor type of stroke.	Experimental group: used a virtual reality-based system delivered via the Internet, which provided motor tasks to the patients from a remote rehabilitation facility. The control group underwent traditional physical therapy for the upper limb. Both treatments were of 4 weeks duration, 1 hour a day, 5 days a week.	Fugl-Meyer Assessment of the upper extremity (FMA-EU) Ashworth assessment scale	Both rehabilitative therapies significantly improved all outcome scores after treatment. Only the Fugl-Meyer Upper Extremity scale showed differences in the comparison between groups.
Rodriguez-de-Pablo et al., (2015)	n=19 (n=11 in experimental group) Adults Subacute stroke No information about stroke severity, nor type of stroke.	Experimental group: used during 38 sessions a robotic system that incorporated a set of games for the assessment of arm function, the Arm Assist Assessment (AAA), which allows a remote monitoring of the progress of the patient and an automatic adaptation of the therapy. Experimental and control groups underwent 3 weeks of therapy, at least 1 hour of conventional therapy five days a week.	Fugl-Meyer Assessment (FMA) Action Research Arm Test (ARAT) Wolf Motor Function Test (WMFT)	Statistically significant correlation could be shown with the three clinical tests between the standard clinical scales and AAA.
Piron et al., (2008)	n=10 (n=5 in experimental group) Adults Ischemic stroke Time after stroke: control group: 13 months; experimental group:10 months Mild to intermediate arm motor impairment	Experimental group underwent a virtual reality (VR) therapy program at home (Tele-VR group). Control group patients underwent the same VR therapy in a hospital setting (VR-group). Both groups used a 3D motion tracking system to create a virtual environment in which the patient's movement was represented.	Satisfaction questionnaire Fugl-Meyer Assessment of the upper extremity (FMA-EU)	Both rehabilitative therapies significantly improved all outcome scores after treatment, but only the Fugl-Meyer Upper Extremity scale showed differences in the comparison between groups. Significant (p≤0.05) improvement in the Fugl-Meyer UE was seen in the TR group; the control group showed no significant change.

The PEDro scale was developed to quickly identify RCTs with internal validity and sufficient statistical information among the uploaded RCTs. The scoring of PEDro scale is significantly influenced by blinding since different types of blinding are scored by three items. Therefore, the quality score of RCTs related to disciplines in which blinding cannot be completely achieved is underestimated with this tool. PEDro total scores above 6 (including 6) were considered high quality (Moseley et al., 2002); scores below 6 were considered fair quality.

According to this tool, the methodological quality of included studies in this systematic review was generally high (Table 3), as four studies scored more than 6 points.

**Table 3:** RCT Quality Assessment Using PEDro Scale

PEDro scale	Choi & Paik, (2018)	Fluet, et al., (2019)	Piron, et al., (2009)	Rodriguez-de-Pablo, et al., (2015)	Pironeet al., (2008)
Eligibility criteria were specified.	Yes	Yes	Yes	Yes	Yes
Subjects were randomly allocated to groups (in a crossover study, subjects were randomly allocated an order in which treatments were received).	Yes	Yes	Yes	NA	Yes
Allocation was concealed.	NA	NA	Yes	NA	NA
The groups were similar at baseline regarding the most important prognostic indicators.	NA	Yes	NA	NA	Yes
There was blinding of all subjects.	Yes	No	No	No	No
There was blinding of all therapists who administered the therapy.	NA	No	Yes	No	NA
There was blinding of all assessors who measured at least one key outcome.	Yes	NA	Yes	NA	Yes
Measures of at least one key outcome were obtained from more than 85% of the subjects initially allocated to groups.	Yes	Yes	Yes	Yes	Yes
All subjects for whom outcome measures were available received the treatment or control condition as allocated or, where this was not the case, data for at least one key outcome was analyzed by “intention to treat.”	Yes	Yes	Yes	Yes	Yes
The results of between-group statistical comparisons are reported for at least one key outcome.	Yes	Yes	Yes	Yes	Yes
The study provides both point measures and measures of variability for at least one key outcome.	Yes	Yes	Yes	Yes	Yes
**Total score**	**8**	**7**	**9**	**5**	**8**

*Note.* NA: information not available or not enough to make conclusions on that item.

Lack of information was a common feature in all studies. Information regarding the most important prognostic indicators was often omitted, as well as information about the initial functionality of the participants' upper limb. The heterogeneity of selection and intervention protocols and outcome measuring (many studies did not specify outcome measures) also weakened comparison between studies.

## CONCLUSIONS AND FUTURE RESEARCH PERSPECTIVES

The primary focus was the quality of research in virtual reality and telerehabilitation when used together in upper limb rehabilitation post stroke. This review used PRISMA guidelines, which was a strength. The findings, along with identified limitations, could be helpful to future investigations on this topic.

Because RCT is considered the gold standard of clinical investigation, some software systems tested in non RCT studies may not have been included, reducing the pool of articles. Future reviews should include more databases, despite the risk of including more technical articles than clinical trials. The exclusion of articles not written in English may contribute to a limited and even biased perspective. Search terms might be refined to reveal more articles. As examples, “upper extremity” (not only “upper limb”) and “home rehabilitation” OR “home exercises” (not only “telerehabilitation.)”

TR could minimize the discontinuity of treatment after hospital discharge and bring stroke survivors closer to health professionals without geographical barriers, empowering survivors to manage their health via interaction with remote rehabilitation professionals. It is desirous that such teams are interdisciplinary and include end-users (i.e., clinicians and patients) and engineers who embrace a human-centred, holistic approach.

With increasing numbers of stroke survivors, cost-effective measures must be taken to improve their Activity and Participation as detailed in the ICF and strive toward holistic outcomes. VR-based serious games have the ability to scale levels of difficulty in the context of enriched environments and promote motivation. While telerehabilitation using serious games after stroke is a promising tool to complement current services, strong evidence of its benefits is still lacking. More RCT studies are needed with larger numbers of participants and narrower selection criteria (to obtain more homogeneous samples).

Based on this literature review, and despite the lack of RCT studies, it appears that serious games are a valid solution for TR that can bring rehabilitation exercises to the patient's home during the chronic phase at a reasonable price empowering them to manage their health while under constant monitoring from physicians.
